# Genomic characterization of *Plasmodium falciparum* genes associated with anti-folate drug resistance and treatment outcomes in eastern India: A molecular surveillance study from 2008 to 2017

**DOI:** 10.3389/fcimb.2022.865814

**Published:** 2022-12-13

**Authors:** Sabyasachi Das, Satyajit Tripathy, Ankita Das, Meenakshi Kumari Sharma, Ayan Nag, Amiya Kumar Hati, Somenath Roy

**Affiliations:** ^1^ Department of Physiology, Faculty of Medicine, Manipal University College Malaysia, Melaka, Malaysia; ^2^ Department of Human Physiology, Vidyasagar University, Midnapore, India; ^3^ Department of Pharmacology, Faculty of Health Sciences, School of Clinical Medicines, University of the Free State, Bloemfontein, South Africa; ^4^ Department of Human Physiology, Raja NL Khan Women’s College, Midnapore, India; ^5^ Department of Medical Entomology, Calcutta School of Tropical Medicine, Kolkata, West Bengal, India

**Keywords:** *Plasmodium falciparum*, antifolate resistance, *pfdhfr* mutation, *pfdhps* mutation, *in vitro* pyrimethamine resistance, *in vitro* sulfadoxine resistance

## Abstract

**Introduction:**

After being used vigorously for the previous two decades to treat P. falciparum, chloroquine and sulfadoxine-pyrimethamine were replaced in 2009 with an artemisinin-based combination therapy (artesunate-sulfadoxine-pyrimethamine) in an effort to combat multidrug-resistant parasites.

**Methods:**

We set out to assess the genetic variants of sulfadoxine-pyrimethamine resistance and the effectiveness of its treatment in eastern India prior to, during, and 6 to 8 years following the introduction of the new pharmacological regime. In 2008-2009, 318 P. falciparum–positive patients got the recommended doses of sulfadoxine-pyrimethamine. We used 379 additional isolates from 2015 to 2017 in addition to the 106 isolates from 2010. All 803 isolates from two study sites underwent in vitro sulfadoxine-pyrimethamine sensitivity testing and genomic characterisation of sulfadoxine-pyrimethamine resistance (pfdhfr and pfdhps).

**Results:**

In Kolkata and Purulia, we observed early treatment failure in 30.7 and 14.4% of patients, respectively, whereas recrudescence was found in 8.1 and 13.4% of patients, respectively, in 2008–2009. In 2017, the proportion of in vitro pyrimethamine and sulfadoxine resistance steadily grew in Kolkata and Purulia despite a single use of sulfadoxine-pyrimethamine. Treatment failures with sulfadoxine-pyrimethamine were linked to quintuple or quadruple pfdhfr- pfdhps mutations (AICII-AGKAT, AICII-AGKAA, AICII-SGKGT, AICII-AGKAA, AICNI-AGKAA) in 2008–2009 (p < 0.001). The subsequent spread of mutant-haplotypes with higher in vitro sulfadoxine-pyrimethamine resistance (p < 0.001), such as the sextuple (dhfr-AIRNI+dhps-AGEAA, dhfr-ANRNL+dhps-AGEAA) and septuple (dhfr-AIRNI+dhps-AGEAT), mutations were observed in 2015-2017.

**Discussion:**

This successive spread of mutations with high in vitro sulfadoxine-pyrimethamine resistance confirmed the progressive increase in antifolate resistance even after an 8-year withdrawal of sulfadoxine-pyrimethamine.

## Introduction

Globally, 241 million confirmed cases of malaria and an estimated 627,000 deaths were reported in 2020. (WHO, 2021). This represents around 14 million more cases in 2020 compared with 2019 and 69,000 more deaths. Ninety-six percent of malaria-related deaths reported worldwide in 2020 came from Africa (WHO, 2021 prior to 2009, Guidelines for Diagnosis and Treatment of Malaria in India, 2009); sulfadoxine-pyrimethamine combination (SP) was frequently used in India as a second-line therapy against chloroquine-resistant *Plasmodium falciparum*. However, the spread of SP-resistant parasites has since made SP less effective (Ahmed et al., 2004). Pyrimethamine (PYR), an antifolate drug, targets the parasites’ purine biosynthesis pathway, namely, the dihydrofolate reductase (*dhfr*) gene, whereas sulfadoxine (SDX) only targets the dihydropteroate synthase (*dhps*) gene, which prevents parasite DNA replication (Foote and Cowman, 1994; Abdul-Ghani et al., 2014; Heinberg and Kirkman, 2015).

Antifolate resistance is solely caused by point mutations in the genes that produce the proteins DHPS and DHFR (Foote and Cowman, 1994; Juma et al., 2019).

*In vivo* SP-efficacy and genome wide association studies from south-east Asia to Africa including Middle East countries clearly suggested that polymorphism in the *pfdhfr* gene especially at codon 108 (Ser-108 to Asn-108) represented the key mutation, whereas additional mutations at codon N51I and C59R conferred high levels of resistance (Juma et al., 2019; Zhao et al., 2020). In addition to the polymorphism in *pfdhps*-A437G, other alterations (436-Ala, 540-Glu, 581-Gly, and 613-thr) increased the level of resistance to sulphonamides (Sharma, 2012; Abdul-Ghani et al., 2013; Yaqoob et al., 2018; Madkhali et al., 2020). After more than two decades of successful SP use in India, reports indicate the appearance of SP-resistant parasites from various Indian regions (Ahmed et al., 2004; Sharma, 2012; Das et al., 2013a). When at least two *pfdhfr* mutations and one *pfdhps* mutation occurred simultaneously, the clinical consequence of SP-resistance was frequently seen (Das et al., 2013a). Except for the north-eastern states of India, artesunate-SP is still the medicine of choice there, despite later reports of SP-resistant parasites in several regions of the country (Das et al., 2013a; Guidelines for Diagnosis and Treatment of Malaria in India, 2014). This study sought to establish a genotype–phenotype correlation before, during, and 6– 8 years after the implementation of new drug policy by evaluating the genetic variations and clinical characteristics of antifolate resistance in a large cohort of eastern Indian population in light of the emergence of SP-resistant parasites.

## Material and methods

### Selection of patients

In two distinct time periods, we conducted the study in eastern India’s malaria-endemic Kolkata and Purulia. The first phase samples were gathered between April 2008 and November 2010 before and around the time that India’s new drug policy went into effect. After 6–8 years of the new national drug policy, we also collected the samples between March 2015 and December 2017 for the second phase. Patients who experienced a fever with headache, shaking, or vomiting within the previous 24 h and those who had not taken any antimalarial medicine during that time were submitted for a malaria test. We used EDTA-coated vacutainer tubes to collect 3 ml of intravenous blood from 4,652 suspected patients in accordance with the Helsinki procedure.

*P. falciparum* mono-infection determined by microscopic analysis of Giemsa-stained thin and thick blood smears, patients with a parasite density of 1,000–10,000 asexual parasites/l blood, and patients without a history of self-medication with antimalarials were the inclusion criteria for our study (Bloland et al., 2003). Through the use of an allelic family-specific nested PCR (MAD20 and K1 for pfmsp-1 and 3D7 Africa and FC27 for pfmsp-2), monoclonal *P. falciparum* infections were further evaluated (Das et al., 2014). Pregnant women, newborns, people with non-falciparum malaria, people with severe complicated malaria, and people with mixed infections (*P. falciparum + P. vivax*) were excluded from the study (Bloland et al., 2003). The study also eliminated any individuals who had used antibiotics or antimalarial medications prior to the diagnosis of *P. falciparum* infection. We obtained each patient’s or the patient’s legal guardian’s written informed permission. The Vidyasagar University Human Ethical-Committee gave its full approval to the experimental design and protocols (VU/HEC 08-012).

### *In vivo* SP treatment

*P. falciparum*– positive patients received standard SP doses under the supervision of a medical officer in accordance with the national guideline, with adults receiving a single oral dose of three tablets containing 1000 mg of SDX and 50 mg of PYR, whereas children received a single dose of 20 mg/kg of SDX and 1 mg/kg of PYR (Guidelines for Diagnosis and Treatment of Malaria in India, 2009). Within their expiration dates, we used the quality-assured medications duly provided by the Ministry of Health and Family Welfare, Government of India (IPCA, LTD). Giemsa-stained thin blood smears from each patient were prepared in triplicate at intervals of 12 h until the parasite became undetectable. Two skilled microscopists examined blood smears. If any discrepancies in the results were detected, a senior microscopist was consulted for confirmation. On days 1, 2, 3, 4, 7, 14, 21, and 28, treatment follow-ups were conducted to assess the patient’s clinical status and assure their safety (Bloland et al., 2003). Additionally, if malaria symptoms returned in the intervals between the scheduled visits, unscheduled follow-ups were conducted. By analyzing the msp1, msp2, and glurp genes using family-specific nested PCR, it was possible to distinguish between reinfection and recrudescence (Das et al., 2013b).

According to WHO guidelines, we divided the therapeutic efficacy into three categories: early treatment failure (ETF), late treatment failure (LTF), and adequate clinical parasitological response (ACPR) (WHO, 2013). Patients who did not respond to SP were given a regular dose of artemether-lumefantrine (six tablets, each containing 40 mg AM and 240 mg LF) as a rescue medication and were then monitored for the following 28 days.

### *In vitro* drug sensitivity assay

After adapting clinical isolates to *in vitro* growth using our standard protocol, the phenotypic parameters of *in vitro* SP-sensitivity were assessed (Das et al., 2013a). Following our standard laboratory protocol, *P. falciparum* clinical isolates were grown and maintained in culture (Das et al., 2014). The culture plates were incubated at 37°C with a 95% relative humidity environment and 5% CO_2_. After 48 h of incubation with SDX and PYR using our routine laboratory procedures, we measured the 50% inhibitory concentrations (IC50) of SP using a hypoxanthine incorporation assay (Das et al., 2014). We used nonlinear probit/logit regression analysis to express the values (Basco and Ringwald, 2000). Stock and dilutions of PYR and SDX were made, respectively, with ethanol and dimethyl sulphoxide (DMSO, 0.1%). The final concentrations ranged from 50 to 25600 nM for both PYR and SDX. We studied each concentration in duplicate. However, we used the PYR-sensitive and SDX-resistant-3D7 strains as control strains and adhered to the established threshold values *of in vitro* SP sensitivity as previously described (Basco and Ringwald, 2000; Das et al., 2012a).

### Genome analyses

We isolated the parasite DNA from parasitized erythrocytes as mentioned earlier (Basco and Ringwald, 2000). Furthermore, we used the *P. falciparum* wild-sequence (AL844509.2) available in GenBank to create the primers for the *pfdhfr* and *pfdhps* genes using the primer design software Primer3. Using nested PCR, followed by restriction fragment length polymorphism and sequence analysis, we evaluated the single nucleotide polymorphisms of the pfdhfr and *pfdhps* genes (Das et al., 2013a). We used an ABI PRISM big-dye terminator cycle sequencing kit and a 3730-xl genetic analyzer to sequence the *pfdhfr* and *pfdhps* amplicons in order to confirm the mutation by reading both the forward and reverse strands. The sequence was translated using the Protein Analysis System proteomic service (http://www.expasy.org), and the translated sequences were aligned using ClustalW2 (http://www.ebi.ac.uk/clustalw).

### Molecular modeling of DHFR protein

We have discovered a novel *pfdhfr* double mutant haplotype. Using the crystal structure of the DHFR protein from *Plasmodium falciparum* (PDB id: 6A2K) as a template, the double mutant DHFR protein’s 3D structure was modeled in Phyre-2 (Kelley et al., 2015). PyMOL was used to superimpose the wild-type and mutant-type proteins to test the model’s accuracy (The PyMOL Molecular Graphics System, Version 1.8 Schrödinger, LLC, 2016; Badawi et al., 2016).

### Statistical analysis

*In vitro* IC_50_ values for PYR and SDX coupled with parasitemia were provided as mean SD data. Fisher’s exact test and regression analysis were used to investigate the relationship between SP treatment efficacies and various genotypes. Through the use of the Fisher’s exact test and the Mann–Whitney *U* test, we evaluated the associations between the IC_50_s of SP and the *dhfr* and *dhps* genotypes. The Kruskal–Wallis test was also used to compare data among more than two groups. The total cure-rate was evaluated using the Kaplan–Meier estimate. The 95% confidence intervals were calculated using the Clopper-Pearson formula. Multivariate logistic regression analysis was used to evaluate the connections between *pfdhfr*, *pfdhps* mutations, and *in vitro* SP resistance. When *p* < 0.05, a statistical difference was deemed significant. With the help of GraphPad software 3.0. In Stat and Origin 6.1, we conducted the statistical analysis.

## Results

### Study population

**Figure 1:**
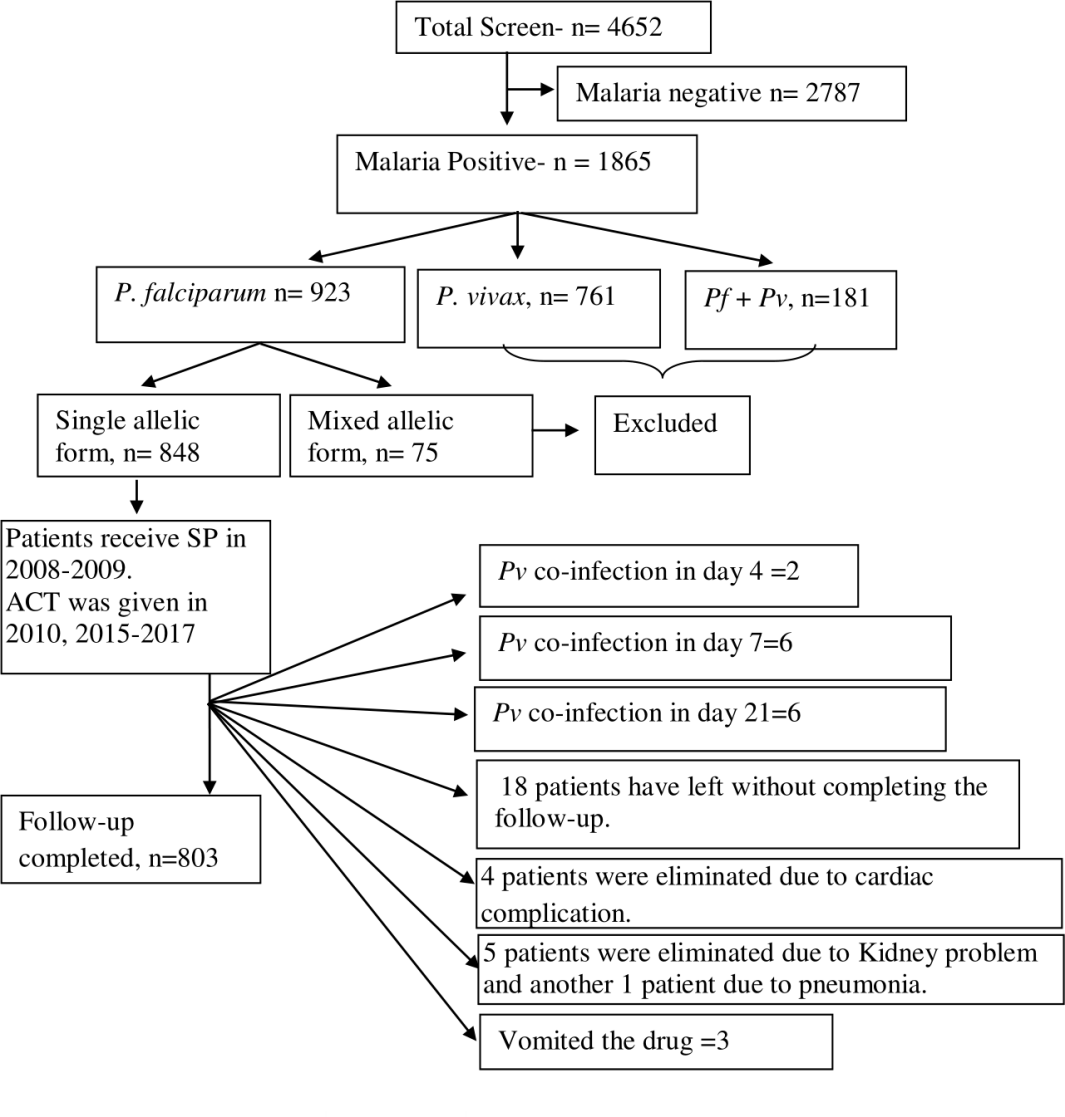
Schematic presentation of patient selection and details inclusion and exclusion criteria for the study. Out of 4,652 screened patients, 2,787 (60.34%) patients were eliminated as they had shown malaria slide negative. A total of 923 patients (19.41%) were found with *P. falciparum* infection. Of them, 75 isolates (8.64%) were identified as mixed *P. falciparum* infection and they were eliminated as these isolates contained both *mspI* and *mspII* alleles. Forty-five patients had not completed the 28-day treatment follow-up, and they were eliminated from the study.

In this investigation, we have paid particular attention to the duration-dependent antifolate susceptibility of *P. falciparum* isolates. Eight hundred three (17.2%) of the 4,652 suspected cases of malaria had finished the 28-day treatment follow-up. These individuals had uncomplicated P*. falciparum* monoclonal infections and had a mean age of 27 years (95% CI: 8–57). Of them, 335 patients were from Purulia, whereas the rest 468 patients were from Kolkata (**Supplementary Table S1**). A total of 761 patients (16.4%) who were *P. vivax*–positive were eliminated. Due to combined P*. falciparum + P. vivax* infection, an additional 181 individuals (3.9%) were removed. While men (57.7%) were found to be more affected in Kolkata, women (53.4%) were found to be more impacted in Purulia. **Table 1** lists the unique traits of participants upon enrolment in the two study locations. Details of patient selection were presented in **Figure 1**.

**Table 1:** Patient characteristics on enrolment of the experiment at different study sites.

Patient Characteristics	Kolkata	Purulia
Age (year)	34.23 (95% CI, 11–57)	31.65 (95% CI, 14–51)
Sex ratio (women/men)	198/270	179/156
Axillary temperature on day 0 (° C)	38.8° C (95% CI, 37.8–40.1)	39.0° C (95% CI, 37.9–40.1)
Parasitemia (parasite/µl)	57128 (95% CI, 19930–93426)	51463 (95% CI, 21500–81429)
Mean haemoglobin (g/dl)	10.4 ± 2.6	9.7± 1.9

### Clinical efficacy of SP

In 2008–2009, 318 P*. falciparum*– infected patients had finished the 28-day SP therapy follow-up. A total of 221 of them were from Kolkata, whereas 97 were from Purulia. A summary of the treatment effectiveness following SP therapy is provided in **Supplementary Table S2**.

In addition, 485 additional *P. falciparum* infections were treated with the artesunate-SP combination in accordance with the national guidelines between 2010 and 2015–2017; however, we were unable to disclose the clinical outcome data due to a lack of sufficient authorization. The genomic characterization of the antifolate resistant genes in those isolates was presented together with each isolate’s unique phenotypic traits of *in vitro* SP susceptibility. However, 205 patients (64.5%) out of 318 P*. falciparum*– infected patients who got SP therapy experienced a successful outcome (ACPR).

More specifically, SP was effective in treating 61.7% of the isolates from Kolkata and 72.1% of the isolates from Purulia. Early treatment failure (ETF) was found in 25.8% of patients based on day 3 positive parasite (parasitemia >1% that of day 0), associated with continuation of fever on day 3, whereas 9.8% of patients were diagnosed as late treatment failure (LTF). Among the study participants, no deaths have been documented. Following the SP treatment, parasite recurrence was seen in three patients on day 4, eight patients on day 7, five patients on day 14, 19 more patients on day 21, and nine more patients on day 28.

All 44 cases of apparent parasite reappearance were subjected to an analysis of merozoite surface proteins 1, 2, and glurp, of which 31 (70.4%) isolates were identified as cases of late SP treatment failure or true recrudescence (**Supplementary Table S2**). Treatment failure for SP in Purulia (8.9% ETF + 8.9% LTF) was recorded in 17.8% of patients in 2008 and increased to 36.5% (19.2% ETF + 17.3% LTF) in 2009. In Kolkata, treatment failure for SP (6.7% ETF + 8.9% LTF) was only recorded in 15.6% of patients in 2008 and increased sharply to 44.9% (36.9% ETF + 7.9% LTF) in 2009. In contrast to Purulia, Kolkata had a higher prevalence of ETF following SP therapy (**Supplementary Table S2**). The overall cure rate following SP therapy was reported to be 61.1% in Kolkata (Kaplan–Meier estimate; 95% CI 54.29–67.48) and 72.16% in Purulia (61.98–80.55). All the treatment failure cases were successfully treated after AMLF therapy.

### *In vitro* SP susceptibility

The phenotypic characteristics of *in vitro* sensitivity for PYR and SDX yielded interpretable results in 795 and 787 isolates, respectively. In Kolkata, the percentage of isolates that were PYR-sensitive in 2008 was 24.4% (mean IC50 = 71.4 nM, 95% CI, 49–91), but it sharply declined to 6.4% by 2017. In Purulia, PYR sensitivity was detected in 46.7% of isolates in 2008 (mean IC50 = 68.8 nM, 95% CI, 51–88 nM), but it dropped to 6.9% in 2017 (mean IC50 = 72 nM, 95% CI, 55–95). In comparison with 2008, the proportion of intermediate PYR resistance significantly decreased at both research locations in 2017. In Purulia, intermediate PYR resistance was just 6.9% in 2017, whereas in Kolkata, intermediate PYR resistance decreased from 28.9% in 2008 to 10.6% in 2017.

**Figure 2:**
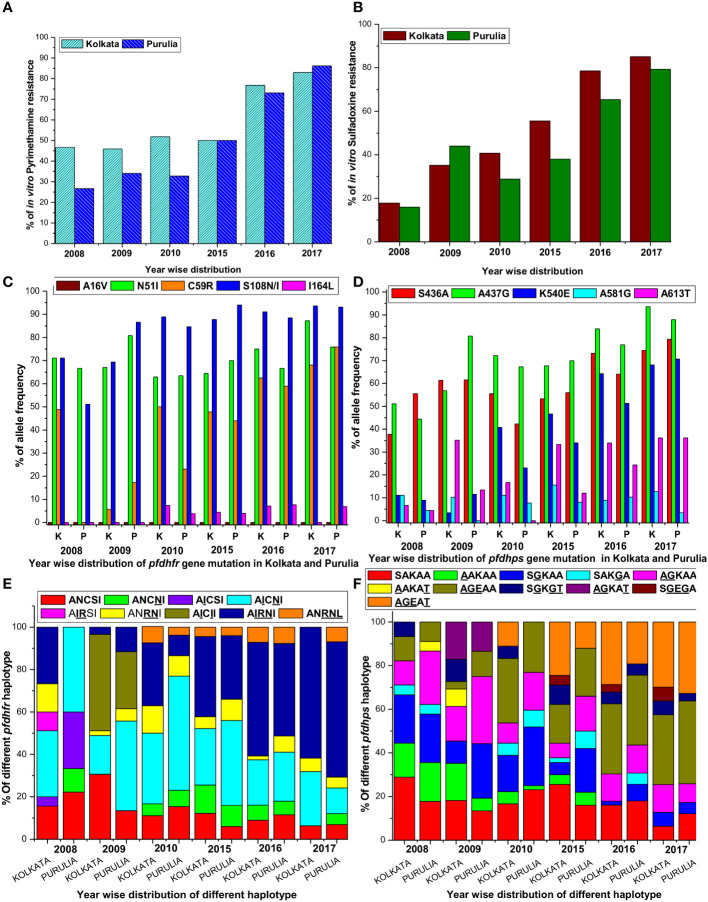
**(A)** Proportion of *in vitro* pyrimethamine resistance from 2008 to 2017 in two study sites. **(B)** Proportion of *in vitro* sulfadoxine resistance from 2008 to 2017 in two study sites. **(C)** Single nucleotide polymorphism of different codons of *pfdhfr* gene in both study sites. **(D)** Single nucleotide polymorphism of different codons of *pfdhps* gene in both study sites. **(E)** Alteration of different *pfdhfr* haplotype frequencies in different period in both the study sites. **(F)** Alteration of different *pfdhps* haplotypes in different time frame at our study sites.

In contrast, in Kolkata, despite no usage of SP since 2009, 83% of isolates were found to be extremely resistant to PYR in 2017 (mean IC50 = 9770.65 nM; 95% CI, 7150–12380), up from 46.7% in 2008 (mean IC50 = 5606 nM; 95% CI, 3155–8168). Additionally, the percentage of PYR-resistance in Purulia gradually grew from only 26.7% in 2008 to as high as 86.2% (mean IC50 = 9120.5 nM; 95% CI, 6075–12065) in 2017 (**Figure 2A**).

In Kolkata, the majority of SDX-sensitive parasites were found to be predominant (53.3%) in 2008 but significantly declined to 6.4% in 2013. In 2008, only 17% of isolates were reported to be SDX resistant (mean IC50 = 6015.35 nM; 95% CI, 3988–8050); however, this number rose dramatically to 85.1% in 2017 (mean IC50 = 10220.5 nM; 95% CI, 7800–12500). In Purulia, 55.6% of isolates were found to be SDX-sensitive in 2008, but this figure had dropped to 12.1% in 2017 (mean IC50 = 351.5 nM, 95% CI, 155–560). SDX resistance was gradually increased to 79.3% in 2017 (mean IC50 = 9670 nM; 95% CI, 6980–12390 nM) from only 15.6% in 2008 (**Figure 2B**). Pyrimethamine and sulfadoxine *in vitro* responses were highly correlated (*r* ^2^ = 0.9965, *p* < 0.019). On the other hand, intermediate SDX resistance in Kolkata fell to 8.5% in 2017 from 29.7% in 2008, whereas intermediate PYR resistance in Purulia was around 8.6% in 2017.

### Genetic architecture of *pfdhfr* and *pfdhps* before new national drug policy

**Figure 3:**
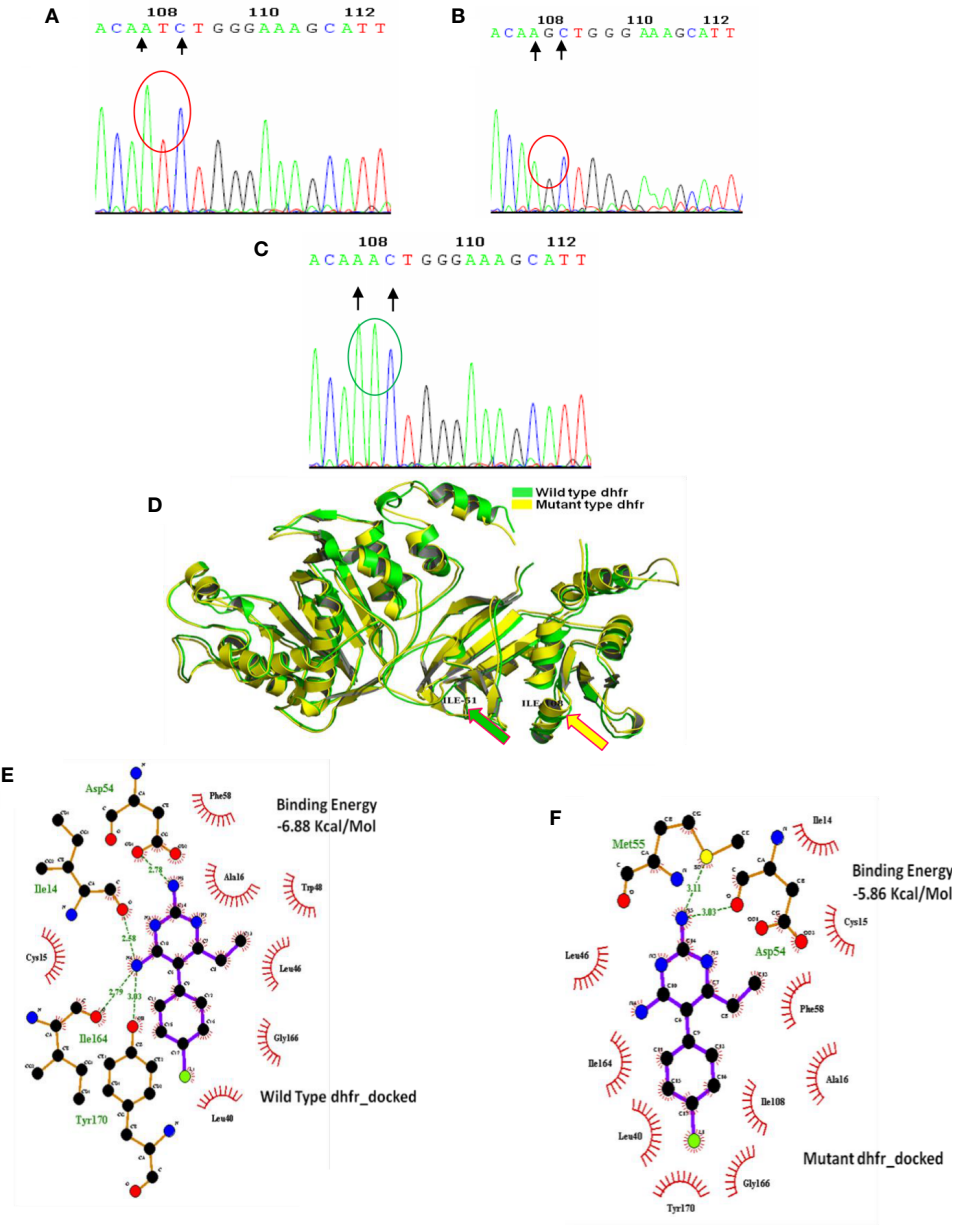
Genomic and proteomic analysis of mutant *dhfr* 108 codon. Through DNA sequence analysis, an alteration of AGC **(B)** to ATC **(A)** was observed at codon 108, which encoded mutant isoleucine (108I) **(A)**. The Expert Protein Analysis System confirmed the presence of isoleucine (ATC). In the wild-type allele, AGC expresses serine at 108 positions of *dhfr* gene, but after mutation, it was replaced with AAC (encoding asparagine) **(C)**. **(D)** Superimposition of wild and mutant allele: Super-imposition of wild and novel double *dhfr* mutation (108I + 51I) proved the uniformity of mutant allele with the wild allele except at 51, and 108 codon and producing a notch having a RMSD of 0·438 Å. **(E)** Molecular docking of pyrimethamine with wild- type *dhfr* gene. **(F)** Molecular docking of pyrimethamine with novel double mutant (*dhfr-*108I+51I) allele. Docking with wild- type *dhfr* protein with pyrimethamine produced high binding energy of 6.88 Kcal/Mole, whereas the docking of double mutant *dhfr* protein with pyrimethamine produced much less binding energy of 5.86 Kcal/Mole. It proves the less affinity of novel mutant *dhfr* protein toward pyrimethamine.

In 2008, 71.1% of Kolkata isolates had the *pfdhfr*-S108N mutation, which was slightly reduced to 69.3% in 2009. In 2009, 45.8% of Kolkata isolates and 26.9% of Purulia isolates encoded novel mutant isoleucine at codon108 (108I) instead of wild serine or mutant asparagine or threonine (KP256234, KP256235, KP256236, and KP256237) (**Figures 3A–C**). This novel *dhfr-*108I allele was always observed with *dhfr*-51I mutation and was only discovered after super-cyclone Aila for 6–8 months. After that, we never saw this haplotype again. The superposition of a novel double mutant (108I + 51I) allele with the wild allele revealed structural uniformity except at the 51 and 108 codons (**Figure 3D**); this resulted in potential conformational changes in the PfDHFR protein, which reduced the binding energy during interaction with pyrimethamine (**Figures 3E, F**), resulting in resistance to pyrimethamine. In 2009, the proportion of Purulia isolates with the most vulnerable S108N or novel S108I mutation increased from 51.1% to 86.5%. In 2008, the N51I mutation predominated in both Kolkata (71.1%) and Purulia (66.7%). In 2009, the N51I mutation was reduced to 67.1% in Kolkata but increased to 80.7 percent in Purulia. In 2008–2009, no mutations were found in the *pfdhfr* gene at codon-A16V and I164L (**Figure 2C**).

In Kolkata, the core *pfdhps*-A437G mutation was found in 51.1% of isolates in 2008 and 56.8% of isolates in 2009, respectively. From 2008 to 2009, Purulia saw a substantial rise in the percentage of isolates with the *pfdhps*-A437G mutation, going from 55.6 to 80.8%. In Kolkata, the prevalence of the S436A mutation significantly rose from 2008 (37.8%) to 2009 (61.4%). In Purulia, the percentage of the mutant S436A-allele was similar in 2008 (55.6%) and 2009 (61.5%). Only 13.5% of the isolates in Purulia had the A613T mutation, compared with a dramatic increase of 35.2% in Kolkata in 2009 (**Figure 2D**).

In Kolkata *pfdhfr-*A **I** C **N** I haplotype was prevalent (31.1%) followed by A **IRN** I (26.7%) and wild ANCSI (15.6%) allele in 2008, whereas in Purulia *pfdhfr-*A **I** C **N** I (40%) was most frequently found haplotype after A **I** CSI (26.7%) and ANCSI (22.2%) allele. In 2009, novel *pfdhfr-*A **I** C **I** I haplotype (45.5%) was predominant followed by ANCSI (30.7%) and A **I** C **N** I allele (18.2%) in Kolkata, while in Purulia, A **I** C **N** I (42.3%) haplotype was commonly found after A **I** C **I** I (26.9%) and ANCSI (13.5%) allele (**Figure 2E**). In Kolkata, wild *pfdhps-*SAKAA haplotype (28.9%) was prevalent after S **G** KAA (22.2%) and **A** AKAA (15.6%) allele whereas, in Purulia, **AG** KAA (24.4%) allele was frequently found after S **G** KAA (22.2%) and **A** AKAA (17.8%) in 2008. Wild-SAKAA allele (18.2%) was prevalent in Kolkata followed by **AG** K **AT** (17.1%), and **A** AKAA allele (17.1) in 2009 whereas, in Purulia, **AG** KAA (30.8%) was most common haplotype after S **G** KAA (25%) (**Figure 2F**).

### Genomic variations of *pfdhfr* and *pfdhps* after new drug policy

Since 2009, no single use of SP has been recommended in India. Instead, in the years that followed, the polymorphism at the *dhfr*-S108N codon grew and peaked in 2017 in Kolkata and Purulia at 93.6% and 931%, respectively. Similar to this, N51I polymorphism grew over the years and peaked in 2017 at 87.2% in Kolkata and 75.9% in Purulia. Following this, the percentage of *pfdhfr*-C59R mutation rose to 68.1% in Kolkata and 75.9% in Purulia. Codon A16V showed no mutations (**Figure 2C**).

In addition, *pfdhps*-S436A and A437G polymorphisms significantly increased from 2010 to 2017 and reached as high as 74.5 and 93.6%, respectively, in Kolkata, whereas 79.3% and 87.9% of isolates, respectively, exhibited mutant-S436A and A437G allele in Purulia. In the following years, the prevalence of the *pfdhps*-K540E mutation quickly rose, reaching as high as 68.1% in Kolkata and 70.7% in Purulia in 2017. The frequency of the *pfdhps*-A581G and A613T mutations did not significantly increase (**Figure 2D**).

Since 2010, wild *pfdhfr*-ANCSI haplotype rapidly reduced in number, whereas most vulnerable triple mutant A **IRN** I allele was predominantly observed in Kolkata (61.7%) and in Purulia (63.8%) followed by double mutant A **I** C **N** I allele (25.5% in Kolkata and 12.1% in Purulia) in 2017. Proportion of single mutant ANC **N** I allele was gradually decreased in subsequent years. Triple mutant AN **RNL** allele was observed for first time in 2010 at both study sites and since then it has maintained a low frequency (< 5%) (**Figure 2E**).

Like *pfdhfr*, frequencies of wild-*pfdhps*-SAKAA haplotype also reduced drastically since 2010. Triple mutant **AGE** AA allele was prevalent in Kolkata (31.9%) and in Purulia (37.9%) in 2017 followed by quadruple mutant **AGE** A **T** allele (29.8% in Kolkata and 32.8% Purulia). Frequency of single mutant S **G** KAA and double mutant **AG** KAA allele were gradually decreased in consequent years. Triple-mutant S **GEG** A allele was observed initially in 2015 at Kolkata and thereafter maintained a very low frequency (<5%) (**Figure 2F**).

### Genotypic association with phenotypes of SP efficacy *in vivo* and *in vitro* during 2008–2009

**Figure 4:**
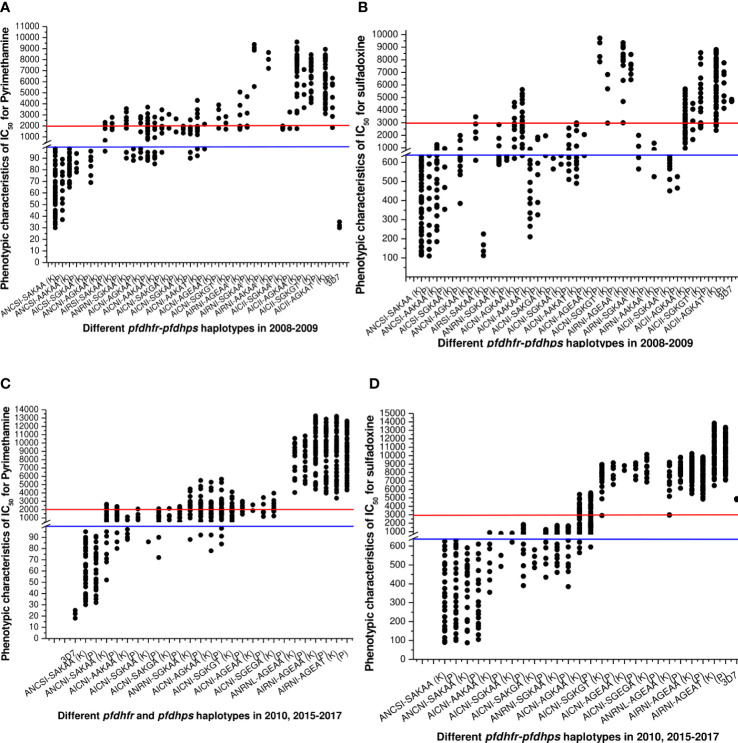
**(A)** Association of *in vitro* pyrimethamine sensitivity (expressed as the IC_50_ of pyrimethamine) with different *pfdhfr* and *pfdhps* haplotype combination before new national drug policy. Here, blue line (corresponding to 100 nM of PYR) was hypothetical showed the sensitive levels whereas red line (corresponding to 2000 nM of PYR) was hypothetical showed the resistance levels for *in vitro* pyrimethamine susceptibility. The isolates presenting *pfdhfr*-ANCSI, ANC **N** I, and AN **RN** I presented low to moderate IC_50_s for PYR and were not associated with *in vitro* PYR resistance (*P* = 0.81, Kruskal–Wallis test) in both study site. **(B)** Association of *in vitro* sulfadoxine sensitivity (expressed as the IC_50_ of sulfadoxine) with different *pfdhfr* and *pfdhps* haplotype combination before new national drug policy. Here, blue line (corresponding to 640 nM of SDX) was hypothetical showed the sensitive levels whereas red line (corresponding to 3000 nM of PYR) was hypothetical showed the resistance levels for *in vitro* pyrimethamine susceptibility. Low IC_50_s for SDX were mostly associated with wild *dhps-*SAKAA allele or single mutant **A** AKAA and S **G** KAA haplotype. ANC **N** I-**AG** KAA, A **I** CSI-S **G** KAA, and ANCSI-**A** AKAA allele presented low to moderate *in vitro* IC_50_ values for SDX and thereby not associated with *in vitro* SDX resistance (*P* = 0.69, Kruskal–Wallis test). **(C)** Haplotype diversity in relation to *in vitro* pyrimethamine susceptibility in 2010, 2015–2017. The phenotype of *in vitro* pyrimethamine-resistance in Kolkata and Purulia was highly correlated with *pfdhfr*-S108N, N51I, and C5159 mutation (*p* < 0.01), but the *pfdhfr* haplotypes were quite different in both study sites. **(D)** Haplotype diversity in relation to *in vitro* sulfadoxine sensitivity in 2010, 2015–2017. In Kolkata *in vitro* sulfadoxine resistance was highly correlated with *pfdhps*-S436A, A437G, K540E, and A613T mutation (*p* < 0.01) but not with 581 mutations whereas in Purulia *in vitro* sulfadoxine resistance was associated with *dhps-*S436A, A437G, and K540E mutations but not with A613T mutation (*p* < 0.05).

In 2008, the number of SP-treatment failure (ETF) cases was very less in both the study sites, but SP-resistant pattern in Purulia was observed differently from Kolkata. In Purulia, ETFs were associated with double *dhfr*-A **I** C **N** I+ triple *dhps-* **AGE** AA mutation (*p* < 0.05) whereas, in Kolkata, isolates representing triple *dhfr*-A **IRN** I +triple *dhps-* **AGE** AA mutation were associated with ETF (*p* < 0.05) in 2008 (**Table 2**). Both haplotype-combinations showed very high IC_50_ for pyrimethamine and sulfadoxine and proved as true *in vitro* resistant isolates (**Figures 4A, B**). In Kolkata, two LTF cases were observed with A **I** C **N** I-**AG** KAA allele. One each LTF case was reported with A **I** C **N** I- S **G** K **GT** and A **IRN** I- **AGE** AA haplotype whereas, in Purulia, one each LTF was found with A **I** C **N** I-**AGE** AA, A **I** C **N** I-**A** AKAT, A **I** C **N** I-SAK **G** A, and ANC **N** I-**AG** KAT allele in 2008. Novel *dhfr*-108I mutation was never observed alone; it was always found with 51I mutation (A **I** C **I** I) and possessed very high IC_50_s for pyrimethamine (*p* < 0.001). In 2009, 64 out of 65 (98%) ETF in Kolkata and seven of 10 (70%) ETF in Purulia represented novel A **I** C **I** I haplotype. ETFs in Kolkata were strongly correlated with A **I** C **I** I-**AG** KA **T**, A **I** C **I** I-**AG** KAA, and A **I** C **I** I-S **G** K **GT** combination (*pfdhfr-pfdhps*) mutations (*p* < 0.001) whereas, in Purulia, ETFs were frequently found with A **I** C **I** I-S **G** K **GT** and A **I** C **I** I-**AG** KAA mutations (*p* < 0.05). Another two ETFs were reported with A **I** C **N** I-**AG** KAA allele in Purulia. On the contrary, in Kolkata, LTFs were frequently found with A **I** C **I** I-**AG** KA **T** and A **IRN** I- **AGE** AA mutations whereas, in Purulia, LTFs were commonly observed with A **IRN** I- **AGE** AA, A **I** C **N** I-**AG** KAA, and A **I** C **I** I-S **G** K **GT** haplotype (**Table 2**). Above mentioned haplotypes represented higher IC_50_s for pyrimethamine and sulfadoxine (*p* < 0.01) (**Figures 4A, B**). In multivariate logistic-regression after adjusting parasitemia, age, and dose of SP, an infection linked with *pfdhfr* A**I**C **I** I-*pfdhps* S **G** K **GT**, *pfdhfr* A**I**C **I** I-*pfdhps* **AG** KAA, and *pfdhfr* A**I**C **I** I- *pfdhps* **AG** KA **T** mutation was substantially more likely to have ETF (odds ratio, 94.4 P < 0.0001).

**Table 2:** Distribution of different *pfdhfr* and *pfdhps* haplotype in relation to *in vivo* SP efficacy and *in vitro* SP susceptibility in Kolkata and Purulia before new national drug policy.

Year	*pfdhfr* haplotype	*Pfdhps* haplotype	No of isolates		SP treatment efficacy						*In vitro* PYR response						*In vitro* SDX response					
	(16, 51, 59, 108, 164)	(436, 437, 540, 581, 613)	K	P	ACPR K	ACPR P	ETF K	ETFP	LTF K	LTFP	S K	S P	IR K	IR P	R K	R P	S K	S P	IR K	IR P	R K	R P
2008	ANCSI	SAKAA	7	6	7	6	–	–	–	–	7	6	–	–	–	–	7	6	–	–	–	–
	ANCSI	**A** AKAA	–	**4**	–	4	–	–	–	–	–	4	–	–	–	–	–	3	–	1	–	–
	**AI** CSI	**S**AKAA	2	2	2	2	–	–	–	–	1	2	1	–	–	–	2	2	–	–	–	–
	A **I** CSI	S **G** KAA	–	10	–	10	–	–	–	–	–	6	–	4	–	–	–	7	–	3	–	–
	ANC **N** I	**AG** KAA	–	**5**	–	4	–	–	–	1	–	1	–	2	–	2	–	1	–	3	–	1
	A **IR** SI	SAKAA	4	–	4	–	–	–	–	–	2	–	2	–	–	–	4	–	–	–	–	–
	A **IRN** I	S **G** KAA	4	**-**	3	–	1	–	–	–	–	–	–	–	4	–	2	–	2	–	–	–
	AN **RN** I	S **G** KAA	6		6								1		5		3		3			
	A **IRN** I	**A** AKAA	3	–	3	–	–	–	–	–	–	–	–	–	3	–	1	–	2	–	–	–
	A **I** C **N** I	**AG** KAA	5	**6**	3	5	–	1	2	–	–	1	2	2	3	3	1	1	2	3	2	2
	A **I** C **N** I	**A** AKAA	4	**4**	4	4	–	–	–	–	1	–	1	2	2	2	3	2	1	2	–	–
	A **I** C **N** I	SAK **G** A	2	**2**	2	1	–	–	–	1	–	–	1	1	1	1	1	2	1	–	–	–
	A **IRN** I	**AGE** AA	5	**-**	2	–	2	–	1	–	–	–	4	–	1	–	–	–	1	–	4	–
	A **I** C **N** I	**A** AKA **T**	–	**2**	–	–	–	1	–	1	–	1	–	–	–	1	–	1	–	1	–	–
	A **I** C **N** I	**AGE** AA	–	**4**	–	1	–	2	–	1	–	–	–	1	–	3	–	–	–	–	–	4
	A **I** C **N** I	S **G** K **GT**	3	**-**	2	–	–	–	1	–	–	–	1	–	2	–	–	–	1	–	2	–
2009`	ANCSI	SAKAA	32	7	32	7	–	–	–	–	28	5	2	–	–	–	32	5	–	–	–	–
	AN **RN** I	S **G** KAA	4	3	4	3	–	–	–	–	4	3	–	–	–	–	2	3	–	–	–	–
	ANCSI	**A** AKAA	22	–	22	–	–	–	–	–	20	–	2	–	–	–	16	–	2	–	–	–
	A **I** C **N** I	S **G** KAA	6	8	5	7	–	–	1	1		3	4	4	–	1	2	4	–	4	–	–
	A **I** C **N** I	**A** AKAA	8	3	8	3	–	–	–	–	6	1	2	2	–	–	8	2	–	1	–	–
	A **I** C **N** I	**AG** KAA	4	11	4	6	–	2	–	3	4	3	–	6	–	2	–	–	3	5	1	6
	A **IRN** I	**AGE** AA	6	6	2	3	1	1	3	2	–	1	4	3	2	2	–	–	–	–	6	6
	A **I** C **N** I	**A** AKA **T**	14	–	12	–	–	–	2	–	2	–	6	–	4	–	6	–	4	–	–	–
	A **I** C **I** I	S **G** KAA	8	2	8	2	–	–	–	–	–	–	8	1	–	1	8	2	–	–	–	–
	A **I** C **I** I	**AG** KAA	24	5	–	1	22	3	2	1	–	–	–	–	24	5	–		11	2	13	3
	A **I** C **I** I	S **G** K **GT**	18	–	–	–	18	–	–	–	–	–	–	–	18	–	–	–	7	–	11	–
	A **I** C **I** I	**AG** KA **T**	30	7	–	1	24	4	6	2	–	–	–	1	30	6	–	–	4		26	7
	**Total**		221	97	135	70	68	14	18	13	75	37	41	29	99	29	98	41	44	25	65	29

Bold and underline amino acid are the mutant codon. Here, in vitro test responses for pyrimethamine are classified as sensitive (S) (IC_50_ value < 100 nM), intermediate resistant (IR) (IC_50_ value 100–2000 nM) and Resistant (R) (IC_50_ value > 2000 nM). In vitro test response for sulfadoxine also classified as sensitive (S) (IC_50_ value < 640 nM), intermediate resistant (IR) (IC_50_ value 640–3000 nM) and Resistant (R) (IC_50_ value >3000 nM). Here, K denotes for Kolkata and P denotes for Purulia.

### Haplotype diversity in association with *in vitro* SP sensitivity after new drug policy

The frequency of wild ANCSI-SAKAA allele subsequently decreased after 2010. The molecular markers associated with *in vitro* SP-resistance were highly varied in two study sites. In Kolkata, *in vitro* PYR resistance was highly correlated to *pfdhfr*-A **IRN** I and AN **RNL** allele *(p* < 0.001), whereas intermediate PYR resistance was associated with A **I** C **N** I haplotype (*p* < 0.001). In Purulia, *in vitro* PYR resistance was strongly associated with *pfdhfr-*AN **RN** I and A **IRN** I haplotype (*p* < 0.01), whereas intermediate PYR-resistance was correlated with ANC **N** I and A **I** C **N** I allele (*p* < 0.001) (**Figure 4C**).

In Kolkata, isolates presenting *pfdhps*-**AGE** A **T,** S **G** K **GT,** and **AGE** AA haplotype possessed very high IC_50_s for sulfadoxine, proving resistant to sulfadoxine (*p* < 0.01). Furthermore, *in vitro* SDX resistance in Purulia was highly correlated with **AGE** A **T** and **AGE** AA haplotype (*p* < 0.01). In both study sites, single mutant *pfdhps*-AAKAA, SAKGA, SGKAA, and double mutant *pfdhps*-AGKAA alleles represented low to moderate IC_50_s for SDX and were associated with intermediate SDX resistant (*p* < 0.05) (**Figure 4D**).

Isolates representing A **IRN** I-**AGE** AA, A **IRN** I-**AGE** A **T**, and AN **RNL**-**AGE** AA combination mutations possessed very high IC_50_s for PYR and SDX and thereby proving highly resistant to PYR and SDX *in vitro* (*p* < 0.001). The isolates containing ANCSI-SAKAA, ANC **N** I-SAKAA, AN **RN** I-S **G** KAA, and A **I** C **N** I-**AG** KAA haplotype represented moderate to high IC_50_s pyrimethamine and sulfadoxine (**Table 3**). In multivariate logistic-regression after adjusting parasitemia, age, and dose of SP, an infection linked with *pfdhfr* A **IRN** I-*pfdhps* **AGE** AA and *pfdhfr* A **IRN** I-*pfdhps* **AGE** A **T** mutation was substantially more likely to have an *in vitro* SP resistance (odds ratio, 89.5, *P* < 0.001; 93.8, *P* < 0.001, respectively).

**Table 3:** *dhfr* and *dhps* haplotypes in relation to *in vitro* SP sensitivity after new drug policy.

Year	*Pfdhfr* haplotype	*Pfdhps* haplotype	No of isolates		*In vitro* PYR response						*In vitro* SDX response					
	16, 51, 59, 108, 164)	(436, 437, 5 40, 581,613)	K	P	S K	S P	IR K	IR P	R K	RP	S K	S P	IR K	IR P	R K	R P
2010	ANCSI	SAKAA	6	8	6	8	–	–	–	–	6	8	–	–	–	–
	ANC **N** I	SAKAA	3	4	1	1	1	2	1	1	3	4	–	–	–	–
	A **I** C **N** I	**A** AKAA	3	1	3	–	–	1	–	–	2	1	1	–	–	–
	A **I** C **N** I	S **G** KAA	2	9	1	3	1	6	–	–	1	4	1	5	–	–
	A **I** C **N** I	**AG** KAA	5	9	1	1	3	5	1	3	1	–	4	6	–	3
	AN **RN** I	S **G** KAA	7	5	1	–	3	2	3	3	5	4	2	1	–	–
	A **I** C **N** I	S **G** K **GT**	3	–	–	–	2	–	1	–	–	–	1	–	2	–
	A **I** C **N** I	SAK **G** A	3	4	–	–	2	3	1	1	2	2	1	2	–	–
	A **I** C **N** I	**AGE** AA	2	5	–	–	1	3	1	2	–	–	–	–	2	5
	A **IRN** I	**AGE** AA	10	5	–	–	–	–	10	5	–	–	1	–	9	5
	AN **RNL**	**AGE** AA	4	2	–	–	–	–	4	2	–	–	1	–	3	2
	A **IRN** I	**AGE** A **T**	6	–	–	–	–	–	6	–	–	–	–	–	6	–
2015	ANCSI	SAKAA	11	3	11	3	–	–	–	–	11	3	–	–	–	–
	ANC **N** I	SAKAA	12	5	5	1	5	3	2	1	12	5	–	–	–	–
	A **I** C **N** I	**A** AKAA	4	3	1	–	3	2	–	1	4	1	–	2	–	–
	AN **RN** I	S **G** KAA	5	5	–	1	2	2	3	2	3	3	2	2	–	–
	A **I** C **N** I	**AG** KAA	6	8	1	1	4	5	1	2	2	1	2	5	2	2
	A **I** C **N** I	SAK **G** A	2	4	–	–	2	3	–	1	2	2	–	2	–	–
	A **I** C **N** I	S **G** KAA	–	5	–	–	–	4	–	1	–	2	–	3	–	–
	A **I** C **N** I	S **G** K **GT**	8	–	–	–	6	–	2	–	–	–	–	–	8	–
	A **IRN** I	**AGE** AA	12	9	–	–	2	–	10	9	–	–	1	–	11	9
	A **IRN** I	**AGE** A **T**	22	6	–	–	–	–	22	6	–	–	–	–	22	6
	AN **RNL**	**AGE** AA	4	2	–	–	–	–	4	2	–	–	1	–	3	2
	A **I** C **N** I	**SGEGA**	4	**-**	–	–	3	–	1	–	–	–	–	–	4	–
2016	ANCSI	SAKAA	5	9	5	9	–	–	–	–	5	9	–	–	–	–
	ANC **N** I	SAKAA	4	5	1	1	2	2	1	2	4	5	–	–	–	–
	A **I** C **N** I	SAK **G** A	–	4	–	–	–	2	–	2	–	1	–	3	–	–
	AN **RN** I	S **G** KAA	1	6	–	–	1	3	–	3	–	–	1	6	–	–
	A **I** C **N** I	**AG** KAA	7	10	–	1	2	2	5	7	–	–	2	3	5	7
	A **I** C **N** I	S **G** K **GT**	3	4	–	–	1	1	2	3	–	–	–	–	3	4
	A **IRN** I	**AGE** AA	14	19	–	–	–	–	14	19	–	–	–	–	14	19
	A **IRN** I	**AGE** A **T**	16	15	–	–	–	–	16	15	–	–	–	–	16	15
	AN **RNL**	**AGE** AA	4	6	–	–	–	–	4	6	–	–	–	–	4	6
	A **I** C **N** I	**SGEGA**	2	**-**	–	–	1	–	1	–	–	–	–	–	2	–
2017	ANCSI	SAKAA	3	4	3	4	–	–	–	–	3	4	–	–	–	–
	ANC **N** I	SAKAA	–	3	–	–	–	2	–	1	–	3	–	–	–	–
	AN **RN** I	S **G** KAA	3	3	–	–	1	–	2	3	–	–	3	3	–	–
	A **I** C **N** I	**AG** KAA	6	5	–	–	2	1	4	4	–	–	1	2	5	3
	A **I** C **N** I	S **G** K **GT**	3	2	–	–	1	1	2	1	–	–	–	–	3	2
	A **IRN** I	**AGE** AA	15	18	–	–	–	–	15	18	–	–	–	–	15	18
	A **IRN** I	**AGE** A **T**	14	19	–	–	–	–	14	19	–	–	–	–	14	19
	AN **RNL**	**AGE** AA	–	4	–	–	–	–	–	4	–	–	–	–	–	4
	A **I** C **N** I	**SGEGA**	3	–	–	–	1	–	2	–	–	–	–	–	3	–
**Sum total**			247	238	40	34	52	55	155	149	66	62	25	45	156	131

Bold and underline amino acid are the mutant codon. Here, in vitro test responses for pyrimethamine are classified as sensitive (S) (IC_50_ value < 100 nM), intermediate resistant (IR) (IC_50_ value 100–2000 nM) and Resistant (R) (IC_50_ value > 2000 nM). In vitro test response for sulfadoxine also classified as sensitive (S) (IC_50_ value < 640nM), intermediate resistant (IR) (IC_50_ value 640–3000 nM) and Resistant (R) (IC_50_ value > 3000 nM). Here, K denotes for Kolkata and P denotes for Purulia.

## Discussion

This study sheds light on the genetic variations and diversity of *pfdhfr* and *pfdhps* haplotypes as well as their relationship to phenotypic characteristics SP-treatment efficacy and *in vitro* SP sensitivity before, at-a-time, and 6–8 years after the implementation of new national drug policy (ACT), allowing us to obtain a thorough understanding of antifolate resistance in eastern India. Although Plasmodium’s genetic diversity was well known, it was still unknown exactly how this diversity affected the clinical manifestation. While there were significantly more LTFs in Purulia than in Kolkata, there were significantly more ETFs in Kolkata. One of the possible explanations for this could be variations in the genetic architecture of *pfdhfr* and *pfdhps*.

In Purulia, triple (double-*dhfr* + single-*dhps*) and quadruple (double-*dhfr* + double-*dhps*) mutations predominated in 2008–2009, but the parasite population in Kolkata exhibited a prevalence of quintuple (double-*dhfr* + triple-*dhps*) and quadruple (double-*dhfr* + double-*dhps*) mutant haplotypes. Changes in SP drug pressure over the population, which were previously seen with *pfcrt* mutations in relation to CQ resistance across the same population, may be the cause of the variation of mutation in two separate areas (Das et al., 2013b). For about two decades, SP was the most commonly given medication in the public sector. This usage may be to blame for the unique selective pressures on the parasite population in Kolkata and Purulia brought on by SP medication. Other factors might be attributed to the uncommon drug resistance situation in West Bengal due to its geographical position and the admixture of inhabitants with multiple tribal ethnic origins. Previous reports from North- East India and Andaman Nicobar Island described quintuple and quadruple mutant *pfdhfr-pfdhps* haplotypes with decreased SP-sensitivity (Das et al., 2010; Das et al., 2012b; Mohapatra et al., 2014). In order to stop the parasite from multiplying, substantially more medicine was needed due to increased mutations that reduced the drug’s ability to connect to the target protein (Yaqoob et al., 2018). We found that isolates with the *pfdhfr*-A **IRN** I, *pfdhps*-**AGE** AA, and *pfdhps*-S **G** K **GT** haplotypes were linked to pyrimethamine and sulfadoxine resistance *in vitro* whereas isolates with the *pfdhfr*-A **I** C **N** I, *pfdhps*-**AG** KAA haplotypes had moderate to high IC_50_s for PYR and SDX.

Following super cyclone Aila in 2009, unique *dhfr*-A **I** C **I** I mutations were found in 48.8% of Kolkata isolates and 26.7% of Purulia isolates, and these alterations were strongly linked with both SP treatment failure and *in vitro* SP resistance. However, the *dhfr*-A **I** C **I** I haplotype was never discovered after 8 months, suggesting that despite its fitness cost, these changes presumably did not benefit the parasite population. Similar to this, the *dhps*-K540N mutation was discovered for the first time at Andaman-Nicobar Island in 2004, following the tsunami; however, it only persisted for 6 months due to a lack of fitness (Lumb et al., 2009).

ACT was introduced in late 2009 in place of SP’s single use in an effort to minimize the prevalence of drug-resistant P falciparum malaria in India. Previous research carried out in Malawi demonstrated that the population’s share of mutant haplotypes was lowered and that the emergence of sensitive parasites was elicited by the discontinuation of a resistant treatment for a period of 8 –10 years. As a result, after 12 years of its discontinuation, CQ-sensitive parasites returned in Malawi (Laufer et al., 2006). However, the proportion of *in vitro* PYR and SDX-resistance was subsequently raised and reached as high as above 85% after 8 years of withdrawal from single use of SP. This was due to steady increases in both study sites in the mutations in the *pfdhfr* and *pfdhps* genes. One of the possible causes of this high SP-pressure could be the extensive and careless usage of SP over the previous two decades (Das et al., 2013a). Additionally, with the exception of the north-east states, SP is one of the sole ingredients in ACT (artesunate-SP) in India. These factors may have recently influenced and increased the pressure of SP-drugs on this parasite population.

The vulnerable *pfdhfr*-164L mutation, which is frequently seen in the surrounding states of Assam and Arunachal Pradesh, has started to develop in both study locations since 2010, which is an alarming sign toward further development of resistant parasite (Sarmah et al., 2017). Despite the single use of SP, emergence and subsequent spread of sextuple (triple-*dhfr*+ triple-*dhps*) (*dhfr*-A **IRN** I +*dhps*-**AGE** AA, *dhfr*-AN **RNL** +*dhps*-**AGE** AA) and septuple (triple-*dhfr* +quadruple-*dhps*) (*dhfr*-A **IRN** I +*dhps*-**AGE** A **T**) mutant haplotypes with severe *in vitro* pyrimethamine and sulfadoxine-resistance confirmed the shift from lower to higher antifolate-resistance in eastern India. The ensuing increase in *pfdhfr-pfdhps* mutations may reduce the effectiveness of artesunate-SP and thus cause the emergence of partial artemisinin resistance, as has previously been observed in eastern India and southeast Asia (Sarmah et al., 2017; Das et al., 2018; Das et al., 2019). Because of the subsequent elevation of pfdhfr-pfdhps mutant haplotypes, it is believed that these mutations happened gradually and step by step (Miotto et al., 2015). The following increase in *dhfr*-A **IRN** I, *dhps*-**AGE** AA, and *dhps*-**AGE** A **T** haplotypes between 2010 and 2017 was the best evidence for this hypothesis (Per se *dhfr*-A **I** C **N** I and *dhfr*-ANC **N** I were much prevalent in 2008–2009). The *pfdhfr* mutation most likely starts with codon-108, additive mutations at codons N51I, C59R, and I164L provide higher degrees of resistance (McCollum et al., 2006; Sandefur et al., 2007). On the other hand, *pfdhps* polymorphism likely starts at codon-A437G and progresses through mutations S436A, K540E, A581G, and A613T. However, as evidenced by the large number of isolates with the *dhps*-**SGEG** A, *dhps*-S **G** K **GT**, and *dhfr*-AN **RNL** haplotypes, the formation of *dhfr* and *dhps* haplotypes did not necessarily occur in an orderly, stepwise fashion.

If gene polymorphism always developed in an orderly, stepwise fashion, then the likelihood of receiving the dhps-613 mutation would only be present in a lineage that already had mutations at codons 437, 436, and 540 (Raman et al., 2010). Similarly, the dhfr-164L mutation would only be found in parasites that already had the 108, 51, and 59 mutations. Our results supported the idea that convergent genotypes could arise in an orderly, stepwise manner, through local parasite evolution, through a *de novo* mutation, or through sequential gene flow as a result of ongoing drug pressure, which eventually contributes to the onset of various *pfdhfr-pfdhps* haplotype variations (Gregson and Plowe, 2005; Karema et al., 2012; Miotto et al., 2015). We lacked certainty regarding genotypic recombination or the accumulation of subsequent point mutations for haplotypes.

The high parasite transmission intensity in India makes it impossible to rule out the possibility of recombination (Sharma, 2012). The sextuple and septuple pfdhfr-pfdhps haplotypes have become stable and common in eastern, north-eastern, and central India despite single use of SP in recent years (Lumb et al., 2009; Mohapatra et al., 2014; Das et al., 2021), showing that these haplotypes are likely benefiting the parasite population without incurring any fitness costs.

In conclusion, the subsequent elevation of *pfdhfr-pfdhps* combination mutation even after 8 years of discontinuing the use of single SP and its correlation with a progressive rise in *in vitro* SP resistance were alarming signs for the country’s efforts to manage and eradicate malaria. In this region of India, antimalarial medications containing SP should not be advised, and the use of alternative antimalarial drug combinations needs to be carefully considered.

## Data availability statement

The datasets presented in this study can be found in online repositories. The names of the repository/repositories and accession number(s) can be found below: https://www.ncbi.nlm.nih.gov/genbank/, KP256234, https://www.ncbi.nlm.nih.gov/genbank/, KP256235, https://www.ncbi.nlm.nih.gov/genbank/, KP256236, https://www.ncbi.nlm.nih.gov/genbank/, KP256237.

## Ethics statement

The studies involving human participants were reviewed and approved by Vidyasagar University Human Ethical-Committee. Written informed consent to participate in this study was provided by the participants’ legal guardian/next of kin.

## Author contributions

SD, AH, and SR contributed to the designing and conceptualization of the manuscript. AH supervised *in vivo* ASSP therapy. *In vivo* data analyses and interpretation were performed by SD, AH, AD, and MS. MS, AD, and AN analyzed the *in vitro* data. SD, ST, AD, AN, and MS contributed to genome analysis, and interpretation of the data. SD, AH, and SR had written the manuscript and gave input at all stages of the study. All authors have approved the final version of the manuscript.
