# Supplementary material for: Genomic characterization of Plasmodium falciparum genes associated with anti-folate drug resistance and treatment outcomes in eastern India: A molecular surveillance study from 2008 to 2017
Source: Front Cell Infect Microbiol. 2022 Dec 13;12:865814. doi: 10.3389/fcimb.2022.865814 (PMC9794033; doi:10.3389/fcimb.2022.865814)
Supplement: Supplementary file 1 [file Table_1.docx]

**Supplementary Table 1: Patients undergoes to SP treatment schedule in Kolkata and Purulia.**

| **Patients completed the SP treatment schedule in 2008-2009** | | | | |
| --- | --- | --- | --- | --- |
| **A). Before new national drug policy** | **Year** | **Kolkata** | **Purulia** | **Total** |
|  | 2008 | 45 | 45 | 90 |
|  | 2009 | 176 | 52 | 228 |
|  | **Total** | | | **318** |
|  | ***in vitro* SP susceptibility in 2010-2013** | | | |
| **B). After new national drug policy** | 2010 | 54 | 52 | 106 |
|  | 2015 | 90 | 50 | 140 |
|  | 2016 | 56 | 78 | 134 |
|  | 2017 | 47 | 58 | 105 |
|  | **Total** | | | 485 |
| **A+B** | **Total** | 468 | 335 | 803 |

**Supplementary Table 2: SP treatment efficacy in Kolkata and Purulia during 2008-2009**

| Year | **ACPR** | | **ETF** | | **LTF (Recrudescence)** | |
| --- | --- | --- | --- | --- | --- | --- |
|  | **Kolkata** | **Purulia** | **Kolkata** | **Purulia** | **Kolkata** | **Purulia** |
| 2008 | 38 (84.44%) | 37  (82.22%) | 3  (6.67%) | 4  (8.89%) | 4  (8.89%) | 4  (8.89%) |
| 2009 | 97 (55.11%) | 33  (63.46%) | 65 (36.93%) | 10  (19.23%) | 14  (7.95%) | 9  (17.31%) |
| **Total** | **135**  **(61.70%)** | **70**  **(72.16%)** | **68**  **(30.77%)** | **14**  **(14.43%)** | **18**  **(8.14%)** | **13**  **(13.40%)** |
| **Grand Total** | **205 (64.46%)** | | **82 (25.79%)** | | **31 (9.75%)** | |
